# Development of a Novel Holistic Assessment Strategy of TAVI-Induced Flow Restoration via Dimensionality Reduction Techniques

**DOI:** 10.1007/s13239-026-00840-2

**Published:** 2026-05-14

**Authors:** Tianai Wang, Tobias Zeus, Christine Quast, Ayse Sedef Ceylan, Kathrin Klein, Malte Kelm, Francesca Dell’Agnello, Simona Celi, Ulrich Steinseifer, Michael Neidlin

**Affiliations:** 1 Cardiovascular Engineering, Applied Medical Engineering, RWTH Aachen University, Forckenbeckstr. 55, 52074 Aachen, Germany; 2 Department of Cardiology, Pulmonary Diseases and Vascular Medicine, Heinrich-Heine University, Düsseldorf, Germany; 3 CARID, Cardiovascular Research Institute Düsseldorf, Heinrich-Heine University, Düsseldorf, Germany; 4 BioCardioLab, Bioengineering Unit, Fondazione Monasterio, Massa, Italy; 5 Department of Information Engineering, University of Pisa, Pisa, Italy

**Keywords:** Aortic flow restoration, Dimensionality reduction, Hemodynamics, Transcatheter aortic valve implantation (TAVI), Proper orthogonal decomposition (POD)

## Abstract

**Purpose:**

Restoration of physiological aortic hemodynamics is a crucial therapeutic target after transcatheter aortic valve implantation (TAVI). However, quantifying this restoration is challenging, particularly using conventional markers. This study presents a novel, unsupervised approach based on proper orthogonal decomposition (POD), suitable for holistic characterization of patient-specific aortic flow field and their association with procedural outcome.

**Material and Methods:**

Patients (n = 8) with severe aortic valve stenosis (AS) undergoing TAVI were studied. Three metrics were evaluated: patient-specific composite cardiovascular stress signature, pre-procedural free hemoglobin levels and degree of flow restoration. Flow restoration was quantified using POD-based similarity measures (Euclidean distances and Dynamic Time Warping), and compared with conventional hemodynamic markers, i.e. shear stresses, helicity and turbulence production. Unsupervised clustering was applied to explore agreement with biochemical patterns and clinical outcomes.

**Results:**

POD identified coherent flow structures and enabled quantification of restoration relative to healthy references. POD-based markers provided more clinically interpretable groupings than conventional measures in this cohort. Patients with low restoration exhibited more pathological composite cardiovascular stress signatures and adverse outcomes during short-term follow-up.

**Conclusion:**

POD-based flow analysis provides a holistic and interpretable quantification of post-TAVI aortic hemodynamics. In the given cohort, the method showed agreement with biochemical patterns and clinical outcomes, highlighting its potential to complement conventional hemodynamic and procedural assessment.

**Supplementary Information:**

The online version contains supplementary material available at 10.1007/s13239-026-00840-2.

## Introduction

Transcatheter aortic valve implantation (TAVI) is a minimally invasive therapy for symptomatic high-risk patients with severe aortic valve stenosis (AS) or asymptomatic patients with severely reduced left ventricular (LV) function or additional adverse prognostic features such as elevated natriuretic peptides [1]. While it leads to an improvement in short-term survival rate and symptoms, it is still associated with post-procedural complications. [2, 3] Further, left ventricular remodeling caused by AS is not always reversed after TAVI, ultimately leading to cardiac dysfunction [4]. Whereas TAVI was previously reserved primarily for high-risk patients, the most recent European Society of Cardiology (ESC) guidelines advocate for an expansion of its indication to include patients at intermediate and low risk [1]. Thus, in order to understand different clinical and anatomical phenotypes, the processes leading to adverse procedural outcomes require further investigations. Moreover, current criteria of AS patient examination and classification can lead to incorrect assignment of patient risk level and risk of overlooking dysfunctional biological processes such as maladaptive LV remodeling [5]. There is increasing interest in evaluating potential ways to predict the risk for adverse outcomes and risk stratification prior to the intervention. Several studies have pointed out an association between certain serum biomarker levels and worse prognosis for AS patients, regardless of symptoms and the traditional indications for TAVI. [6] Redfors et al. emphasize the importance of evaluating the multitude of biomarkers characterizing the biological pathways that are involved in AS onset and progression as a highly heterogeneous disease, even before symptoms become evident [5]. According to Lindman et al., accumulation of multiple of these elevated markers is associated with overall mortality risk post-TAVI [7]. The ESC has likewise acknowledged the relevance of assessing the cumulative burden of multiple elevated biomarkers of cardiovascular stress [1]. Therefore, a comprehensive risk stratification of predictive value should include the complexity of biological processes during AS.

Moreover, AS is associated with strongly altered hemodynamics. Garg et al. (2024) emphasize the complex and highly unique flow patterns in the aortic arch with helical characteristics, which are beneficial for several physiological processes such as vessel perfusion, flow stabilization, etc. In turn, abnormalities trigger subsequent pathological processes such as inflammation, endothelial changes, etc. [8] In a recent study, Quast et al. discovered elevated levels of plasma free hemoglobin (fHb) and consecutive endothelial dysfunction due to the altered flow conditions during AS, which can be partially reversed by TAVI [9]. This study underlines the crucial role of aortic hemodynamic state and changes in AS and TAVI. Given this close relationship between aortic pathology, treatment and hemodynamics, Garg et al. introduce the restoration of aortic flow-related characteristics through intervention as a potential novel therapeutic target in future treatment planning and development [8]. Candidate flow markers are, for instance, flow vorticity and helicity, wall shear stresses (WSS) and systolic flow displacement, which can be derived from in-vivo imaging modalities such as cardiac 4D Flow MRI [8]. Komoriyama et al. examined the effect of TAVI on such metrics, i.e. helicity, vorticity, eccentricity and WSS [4]. Although these conventional markers have potential to describe flow restoration, they suffer from several limitations: In order to evaluate their value, regions of interest need to be chosen arbitrarily and operator dependently, where time and location-restricted probes of the markers are extracted, leading to a simplification from 3D to 2D. Similarly, transient flows require the calculation of time-averaged metrics. Additionally, thresholds (e.g. for WSS) need to be defined to judge whether they fall within a pathological or physiological range. Lastly, low resolution of the data might obscure these derived markers more severely than the primary velocity field, e.g. WSS as a gradient-based quantity or vorticity defined as the curl of velocity. In the case of image-based numerical modeling of vascular flow, these derived parameters show significant additional sensitivity to the choice boundary conditions [10, 11].

Thus, other approaches that do not suffer from these limitations are desirable. Optimally, these approaches quantify the holistic, four-dimensional flow field and take the dynamical behavior of the entire system domain into account [12]. This is especially crucial when the link between flow and disease has yet to be elucidated. In general fluid dynamics, so-called dimensionality reduction (DR) techniques are used as a key tool to analyze high-dimensional data and to recognize data-governing patterns. By orthogonal linear transformation of the physical space into a modal basis, dominant features and information can be extracted and simplified into lower-dimensional representations, while maintaining the relation between data points and structure. [13–15] Proper Orthogonal Decomposition (POD) is a widely used example of such DR techniques, first introduced by Lumley et al. [16]. Later, Sirovich et al. developed the so-called snapshot POD, a data-driven implementation of POD on a distinct time series based on singular value decomposition [17].

In recent years, POD has also entered the field of vascular flow research. Grinberg et al. first applied POD in order to detect and characterize turbulence in numerical stenosed carotid arterial bifurcation models [18]. Complementary to the use of POD for feature extraction, the resulting decomposed modes can also serve as building blocks for Reduced Order Models (ROMs) as reconstructions of complex velocity fields. This was done by Chatpattanasiri et al. for Particle Image Velocimetry and Computational Fluid Dynamics (CFD) data of a patient-specific aortic dissection and utilized as a validation tool of the numerical model [19]. The core strength of POD to extract the most dominant features, i.e. capturing the majority of kinetic energy within the system, of a spatiotemporal flow field was exploited in further studies by comparing the eigenvalues of the POD modes of different vascular flow fields [12, 20]. Finally, Borja et al. carried out modal decomposition of healthy and pathological ventricular flow and built cohort-representative models with characteristic flow metrics based on the resulting POD modes. Subsequent unsupervised classification of patient-specific ventricular ROMs according to these ROM-based phenotypes showed the potential use of POD to discriminate pathology states. [21] However, the promising DR technique has not yet been used to comprehensively assess pathological and post-procedural hemodynamics to a healthy baseline, and therefore to assess the degree of flow restoration through cardiovascular intervention. Further, a systematic comparison to conventional flow analysis parameters has yet to be carried out to assess its capability to comprehensively describe and analyze complex flow fields.

Therefore, the goal of this present study is to present an approach to quantify aortic flow restoration in TAVI to facilitate further association with patient-specific composite cardiovascular stress signatures and known procedural outcomes. This is achieved by developing a novel, POD-based flow analysis approach to holistically describe the aortic flow field. In summary, a systematic link between patient-specific flow restoration, known procedural outcome and individual composite cardiovascular stress signatures is formed.

## Materials and Methods

**Fig. 1:**
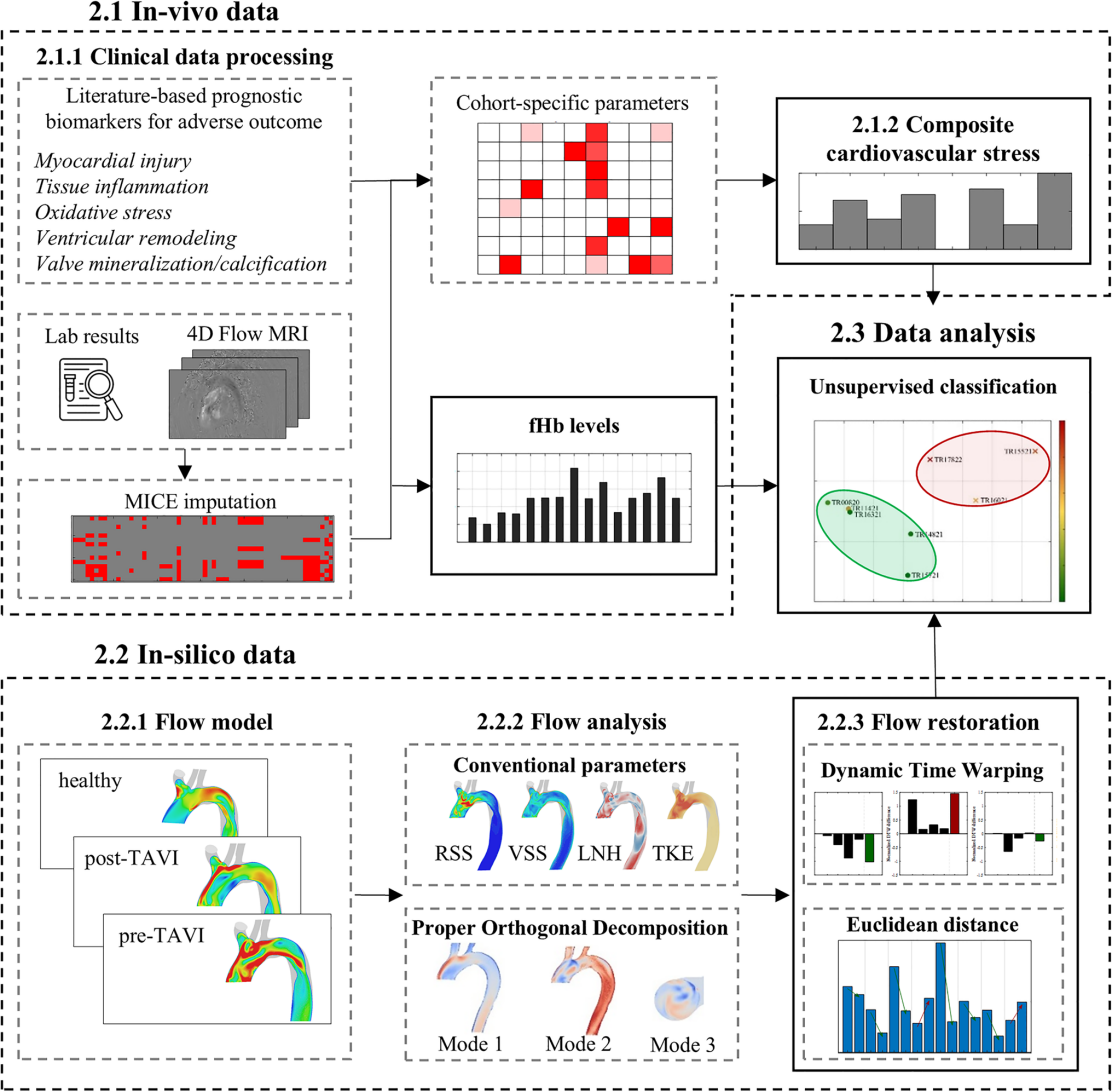
Overview of entire methodological workflow, referring to corresponding subchapters. TAVI: Transcatheter aortic valve implantation; MICE: Multivariate Imputation by Chained Equation; fHb: plasma-free hemoglobin

To investigate the role of hemodynamic restoration following TAVI, three primary metrics were defined and analyzed within a clinical cohort of patients diagnosed with severe AS: a patient-specific composite cardiovascular stress signature, pre-TAVI fHb levels and the degree of flow restoration, assessed using both conventional and POD-based hemodynamic markers. Using unsupervised classification techniques, patterns of association among these descriptors were explored. An overview of the entire methodological workflow is given in Fig. 1. All codes utilized and developed within this study were implemented in MATLAB R2022a (MathWorks, Natick, MA).

### In-vivo Data

The clinical study cohort of n = 15 patients (8 male, 7 female) had a mean age of 79.2 ± 5.6 years, with a mean body mass index (BMI) of 24.9 ± 2.5. All patients were diagnosed with severe AS and underwent a TAVI procedure. 4D Flow MRI scans were taken within one week before and after the intervention, in addition to further medical imaging procedures and routine laboratory measurements. For more details, see Quast et al. [9]. One pre-procedural CT scan was undertaken for each patient. All measurements and scans were originally acquired at the University Hospital Düsseldorf (UKD), anonymized and made available for retrospective analysis. Approvals were granted for all acquisitions by the Institutional Review Board and all participants provided written informed consent. 4D Flow MRI data were acquired on 1.5 T MRI systems (Achieva, Philips Healthcare, The Netherlands) using the torso and posterior coil and followed general guidelines and recommendations according to the consensus statement by Dyverfeldt, Bissell et al. [22]. Single source CT data were acquired using a SOMATOM Definition AS + (Siemens Healthcare, Forchheim, Germany), with a temporal resolution of 150 ms and collimation of 128 x 0.6 mm.

#### Clinical Data Processing

From the initial cohort of n = 15 patients, a final sub-cohort of n = 8 patients (5 male, 3 female) was retained based of predefined technical inclusion criteria required for the applied workflow. Patients were excluded if (i) the 4D Flow MRI scans did not fully cover the region of interest, particularly the supra-aortic vessels required for consistent domain definition, (ii) the 4D Flow MRI exhibited substantial image-quality limitations such as extended aliasing regions, or (iii) required biochemical measurements were too scarce. To address incomplete clinical data entries, missing values were imputed using the Multivariate Imputation by Chained Equations (MICE) method. If more than 40 % of data were missing, the corresponding feature or patient was removed from the subsequent analyses. These inclusion criteria were independent of clinical outcome or hemodynamic result, minimizing risk of bias. The sub-cohort had a mean age of 77.4 ± 4.8 years and an average BMI of 23.9 ± 1.4. A detailed overview of relevant clinical documentation of these patients, including pre-procedural surgical risk assessments, prosthesis implantation quality and metrics, and post-procedural adverse outcomes (30 days post-TAVI), can be found in Supplementary Material S-I.

##### fHb Levels

Following imputation, the first metric derived for analysis was the pre-procedural plasma free hemoglobin level (fHb) obtained before intervention. Elevated fHb reflects subclinical hemolysis induced by altered mechanical shear stresses and disturbed flow behavior and thus provides a flow-related indicator of valvular disease burden It was evaluated independently of further parameters, due to its direct mechanistic link between altered supravalvular hemodynamics and biological response in aortic stenosis [9]. 

##### Prognostic Biomarkers for Adverse Outcomes

A portion of the biomarkers with prognostic value in the context of AS and TAVI outcome were also included in the clinical dataset of this study. These biomarkers reflect systemic inflammation, lipid metabolism, and immune modulation, which are processes that are increasingly recognized as contributing to both valvular degeneration and cardiovascular risk in this patient population. The selected biomarkers and their roles are briefly outlined below, according to the reviews provided by Redfors et al. [5] and Sarkar et al. [6]:

- C-reactive protein (CRP): As a sensitive marker of systemic inflammation, elevated CRP levels have been associated with accelerated valvular calcification and poorer outcomes following TAVI.
- High-density lipoprotein (HDL): Known for its protective cardiovascular effects, lower HDL levels correlate with increased atherosclerotic burden and systemic inflammation in AS patients. Especially the combination of low baseline HDL and high CRP has shown to be predictive for higher mortality post-TAVI.
- Low-density lipoprotein (LDL): Elevated LDL is implicated in lipid infiltration and calcific processes at the valve level. Low LDL is associated with adverse events in the cardiac and cerebrovascular system post-TAVI and overall poorer AS outcome.
- Total cholesterol: Serving as a general marker of lipid metabolism and cardiovascular risk, a significant link between the cholesterol levels and adverse cardiac events in patients with AS was identified.
- Lipoprotein (a) (Lp(a)): Similarly to LDL, LP(a) is linked to valve level lipid infiltration due to endothelial damage. Significant positive correlation between Lp(a) and AS progression was shown.
- Relative monocyte count: Monocytes play a key role in inflammatory and fibrotic responses; higher levels may indicate ongoing immune activation relevant to both valve degeneration and post-TAVI recovery. In particular, if a high monocyte count coincides with high baseline levels of CRP and IL-6, the all-cause mortality risk increases.
- Interleukin-6 (IL-6): A pro-inflammatory cytokine involved in immune signaling, IL-6 is elevated in chronic inflammatory states and may predict adverse outcomes after TAVI.
- CD4/CD8 ratio: As marker of immune system balance, this ratio reflects the balance of helper and cytotoxic T cells. A decrease in CD4/CD8 ratio has been associated with increased all-cause 1 year mortality and overall poorer functional recovery.
- NT-proBNP: Reflective of myocardial stress and heart failure severity, baseline NT-proBNP levels have shown to predict symptom response to TAVI, correlating with poor functional outcomes at 1 year. Persistently elevated levels can indicate higher mortality risk post-TAVI.

Since both the number of elevated or altered biomarkers and their individual elevation degree or magnitude are associated with post-procedural complications and overall poorer outcome post-TAVI, a composite cardiovascular stress signature was defined for each patient.

#### Composite Cardiovascular Stress Signature: Definition and Calculation

A composite cardiovascular stress signature was derived to reflect the cumulative burden of pathologically altered biochemical parameters in the pre-interventional state.

- Identification of abnormal values: For each patient, the relevant biochemical parameters were assessed and classified as pathologically elevated or altered based on established clinical thresholds or literature-derived reference ranges.
- Quantification of relative deviation: The degree of deviation from physiological values was quantified for each parameter, scaled relative to the distribution within the study cohort.
- Aggregation: Individual parameter deviations were summed to generate an aggregated value per patient, representing a cumulative burden of cardiovascular stress described in prior clinical studies and current ESC guidelines [1].
- Normalization: The aggregated values were normalized across the cohort to yield a unitless metric ranging from 0 to 1, facilitating inter-patient comparison. A value of 1 indicates a higher burden of pathological deviations, whereas 0 indicates a more physiological pre-interventional pattern.

This composite cardiovascular stress signature is not an established clinical tool and is not intended for clinical decision-making or statistical inference. Rather, it is introduced as an exploratory, literature-informed descriptor to provide clinical context for the proposed hemodynamic analysis.

### In-silico Data

Patient-specific, 4D Flow MRI based CFD models were generated for the sub-cohort of n = 8 subjects using the commercially available CFD software package ANSYS CFX 2024 R1 (Canonsburg, PA), including the solid modeling software ANSYS Spaceclaim 2024 R2, and the meshing tool ICEM CFD 2024 R2.

#### Flow Model: 4D Flow MRI-Based CFD

The numerical models were developed for the pre- and post-TAVI state for each patient, following the methodology previously outlined in Wang et al. [23]. Patient-specific geometries were segmented from in-vivo CT data, employing a previously described 3D UNet model [24]. The computational domain comprised the ascending aorta, aortic arch including the supra-aortic branches up to the first bifurcation level, and the thoracic descending aorta. The domain did not include the native valve, TAVI prosthesis, aortic root, or coronary arteries explicitly. This modeling choice was made since the endpoint of interest in the present study was the downstream thoracic flow field rather than leaflet kinematics, sinus or root flow, or coronary perfusion. The segmentations were meshed using unstructured tetrahedral meshes and 12 prism layers at vessel wall. The pre- and post-TAVI models of a patient shared the same geometry, mesh, and outlet boundary conditions, while the inflow boundary conditions were state-specific. Inflow boundary conditions were defined by 4D Flow MRI-derived supra-valvular velocity profiles. Because the post-TAVI 4D Flow MRI was acquired immediately after intervention, relevant vascular remodeling was assumed negligible, and the hemodynamic impact of the altered valvular outflow was represented through the corresponding measured supra-valvular inflow profile. Outlet boundary conditions were prescribed using branch-specific three-element Windkessel (RCR) models for the descending aorta and all supra-aortic branches, with literature-based parameter values following Pirola et al. [11]. These parameters approximate the downstream vasculature and preserve a physiological flow split between the outlets.

Blood flow was modeled using the non-Newtonian Carreau–Yasuda model. The Reynolds-Averaged Shear Stress Transport (SST) model was used consistently across all cases, with an inlet turbulence intensity of 5%, to account for transitional and disturbed flow features, particularly in AS, and to maintain comparable modeling assumptions between states.

Additionally, for each patient, a synthetic healthy inflow profile was defined. A parabolic axial velocity profile was scaled such that its instantaneous volumetric flow rate matched the 4D Flow MRI-derived flow waveform of the corresponding patient. Secondary in-plane velocity components were then derived from the measured 4D Flow MRI data by calculating their time-resolved relative magnitude with respect to the axial component and multiplying these scaling factors with the synthetic axial velocity. Following this approach, the healthy reference preserved patient-specific secondary flow characteristics while reducing the influence of pathologies. The corresponding synthetic healthy CFD models served as patient-specific healthy baseline flow.

Transient simulations were performed using a timestep equal to 1/20 of the temporal resolution of the 4D Flow MRI data, resulting in physical timesteps of approximately 0.035 to 0.045s. Each simulation was simulated over three cardiac cycles, with up to ten inner coefficient iterations per timestep. Numerical convergence was monitored by maintaining a root-mean-square residual below 10^-4^ for both mass and momentum equations. The advection term was discretized using a Specified Blend Factor of 0.75 and temporal discretization was achieved with a second-order Backward Euler scheme. To account for influence of initial transient effects, the third simulated cardiac cycle was considered for analysis. For further implementation details, the reader is referred to Wang et al. [23].

#### Flow Analysis: Conventional Parameters and Proper Orthogonal Decomposition

To analyze the resulting fields, two approaches were implemented: First, conventional hemodynamic parameters of interest were extracted and assessed as time-averaged metrics in the aortic domain. Next, the most dominant flow features were extracted from the spatiotemporal velocity fields by applying mean-centered POD.

##### Conventional Parameters

The parameters of interest represent broadly used hemodynamic features, particularly influential for the extent of blood cell damage. Based on the resolved flow field, their instantaneous values were calculated as explained in the Supplementary Material S-II.

According to Quast et al. [9] and Wang et al. [23], the primary contributor to sub-hemolytic effects on red blood cells in AS is the alteration of bulk flow, particularly the resulting increase in shear stress. Consequently, scalar shear stress metrics were assessed. These included viscous shear stresses (VSS), quantifying the shear strain responsible for cell deformation, Reynolds shear stresses (RSS), correlating with elevated risk for cellular damage, and total shear stress (TSS) as an aggregate descriptor of overall flow-induced stress exposure [25, 26]. Due to the swirling and helical nature of flow in the aorta, helicity is a widely employed parameter to characterize aortic hemodynamics, thus, holding potential to be used for diagnostic or prognostic purposes [27]. Elevated helicity is often observed in aortic valve disease and is studied in the context of cardiovascular pathologies and flow efficiency [27]. In this study, its normalized form, the Local Normalized Helicity (LNH), was assessed. LNH was used by Garcia et al. to discriminate between patients with aortic dilation and a healthy control group [28]. Finally, Wu et al. formulated a relationship between hemolysis and energy dissipation within the energy cascade from large scale turbulent motion to small eddies [29]. Since AS is characterized by a pathological increase in turbulent flow structures, turbulence kinetic energy (TKE) was included as an additional metric of interest. TKE is particularly useful for analyzing pathological blood flows, as it is independent of the resolution of the in-vivo measurements. They particularly recommended its use in the context of AS [30]. 

##### POD-Derived Parameters

POD is an application of singular value decomposition to a set of time resolved data, yielding an orthogonal modal basis that represents the data with minimized mean squared reconstruction error. This allows for the extraction of dominant spatial modes with decreasing energy contribution to the overall energy of the system. When applied to a spatiotemporally resolved velocity field, these modes represent coherent flow structures within the flow. In turn, these modes can be utilized for ROM where the original complex system is represented by a linear combination of these dominant modes, capturing sufficiently large portions of the system energy [13]. 

In this study, the method of mean-centered snapshot POD, introduced by Sirovich [17], was applied. Given a 3D scalar field, representing the three-directional velocity on a spatial grid of N points at M time steps, the procedure can be summarized as follows [19, 21]:

1. Construction of the Snapshot Matrix U
  The temporal mean velocity $\overline{{{\boldsymbol{u}}\left( {x,y,z} \right)}}$ is subtracted from each instantaneous velocity field ${\boldsymbol{u}}_{i}$ at time instance *i*. This results in the instantaneous fluctuating velocity field ${\boldsymbol{u}}_{i} \boldsymbol{^{\prime}}\left( {x,y,z} \right)$ around the time-averaged velocity. By rearranging each velocity field into a single column vector and concatenating them to a joined matrix, the snapshot matrix **U** is obtained:
  1 $${\boldsymbol{U}} = \left[ {{\boldsymbol{u}}_{1}^{\boldsymbol{^{\prime}}} ,{\boldsymbol{u}}_{2}^{\boldsymbol{^{\prime}}} , \ldots ,{\boldsymbol{u}}_{M}^{\boldsymbol{^{\prime}}} } \right] = \left( {\begin{array}{*{20}c} {u^{\prime}_{1,1} } & {u^{\prime}_{1,2} } & \cdots & {u^{\prime}_{1,M} } \\ \vdots & \vdots & \ddots & \vdots \\ {u^{\prime}_{N,1} } & {u^{\prime}_{N,2} } & \cdots & {u^{\prime}_{N,M} } \\ {v^{\prime}_{1,1} } & {v^{\prime}_{1,2} } & \cdots & {v^{\prime}_{1,M} } \\ \vdots & \vdots & \ddots & \vdots \\ {v^{\prime}_{N,1} } & {v^{\prime}_{N,2} } & \cdots & {v^{\prime}_{N,M} } \\ {w^{\prime}_{1,1} } & {w^{\prime}_{1,2} } & \cdots & {w^{\prime}_{1,M} } \\ \vdots & \vdots & \ddots & \vdots \\ {w^{\prime}_{N,1} } & {w^{\prime}_{N,2} } & \cdots & {w^{\prime}_{N,M} } \\ \end{array} } \right)$$
  Each column of **U** then contains the entire flow field at one time instance. Therefore, the number of columns in **U** is equal to the number of time steps.
2. Application of Singular Value Decomposition (SVD)
  The SVD of **U** is defined as:
  2 $${\boldsymbol{U}} = \boldsymbol{ \Phi \Sigma \Psi }^{\boldsymbol{*}}$$
  With ${{\boldsymbol{\Phi}}}$ being the left singular matrix, where the columns are orthogonal eigenvectors representing the POD modes, and ${{\boldsymbol{\Psi}}}^{\boldsymbol{*}}$ the conjugate transpose of the right singular matrix of **U.** ${{\boldsymbol{\Sigma}}}$ is the singular diagonal matrix with singular values $\sigma_{i}$ on the diagonal axis. The singular values quantify the energy content $\sigma_{i}^{2}$ of each corresponding mode ${{\boldsymbol{\Phi}}}_{{\boldsymbol{i}}}$ and are sorted in descending order of their energy amount. Thus, the first mode represents the largest portion of the system’s energy. In total, the maximum amount of resulting modes M is equal to the number of discrete time steps T_max_ included in the decomposition.
3. Calculation of temporal POD coefficients *b*_*i,t*_
  Finally, **U** can be approximated as a linear combination of the POD modes with the temporal coefficients ***b***_*i*_*(t):*
  3 $${\boldsymbol{U}}\left( t \right) \approx \overline{\boldsymbol{u}} + \mathop \sum \limits_{i = 1}^{M} b_{i} \left( t \right){\boldsymbol{\varPhi}}_{{\boldsymbol{i}}}$$
  This mean-centered snapshot POD method was applied to all numerical aortic flow fields for the three states of the sub-cohort of n = 8 patients (pre-TAVI, post-TAVI, healthy), resulting in 8x3 POD applications. Each POD mode and their corresponding temporal coefficient represent a specific flow feature and its contribution to the overall system energy. The most dominant flow features, or the most energetic POD modes, which contain more than 90 % of the total energy, were plotted, analyzed and compared between the models. Further, their ability to reconstruct the original flow field is verified within an accepted range of reconstruction errors.

#### Flow Restoration: Dynamic Time Warping and Euclidean Distances

The main motivation of this study was to quantify the similarity of a pre/post procedural aortic flow field to a (synthetic) healthy flow. We therefore defined the flow restoration degree by comparing the hemodynamic similarity of pre/post states state to the metrics of the corresponding synthetic healthy baseline model for each patient, respectively.

##### Euclidean Distance: Conventional Parameters

For each conventional parameter, the Euclidean distance was calculated independently without aggregation. Specifically, for a given instantaneous 3D scalar field of a given parameter *X*, the cycle-averaged scalar field was evaluated over all spatial grid points in the domain and compared between point clouds of the healthy reference and the corresponding pre- or post-TAVI state. In this study, the entire 3D aortic arch was evaluated to avoid bias from preselecting regions based on prior knowledge, which is a limiting aspect when assessing conventional parameters. This resulted in two Euclidean distance values *D*_*pre/post*_ per parameter:

4 $$D_{pre/post} = \sqrt {\frac{1}{N}\mathop \sum \limits_{i = 1}^{N} \left( {X_{i}^{pre/post} - X_{i}^{healthy} } \right)^{2} }$$

The final conventional restoration metric was then defined as the change in Euclidean distance between pre- and post-TAVI relative to the healthy reference, i.e. *D*_*Eucl-conv*_* = D*_*post*_* - D*_*pre*_*.* This metric is representative of the similarity of the time-averaged scalar fields of the altered pre- or post-procedural state to the physiological case.

##### Euclidean Distance: POD-Derived Parameters

Subsequently, time-averaged, normalized POD weights $B_{r}$ for the first R modes *r = 1,...,R* per patient and state were calculated from the temporal POD coefficients ***b***_***r***_*(t)* as follows [21]:

5 $$B_{r} = \frac{{\smallint b_{r}^{2} dt}}{{\mathop \sum \nolimits_{j = 1}^{M} \smallint b_{j}^{2} dt}}, r = 1, \ldots , R$$

R is defined as the mode number, where the summation of kinetic energies of all modes up to R capture more than 90% of the total system energy content. By concatenating the scalar weight values for mode *r = 1,…,R* for each patient and case, an *Rx1* vector ***B*** is obtained per patient and case, where each entry describes the normalized contribution of the POD mode r to the overall flow field.

Subsequently, the Euclidean distance ***D***_***Eucl-POD***_ between the weight vector ***B*** for the pre- and post-TAVI case to the ***B*** vector of the healthy baseline was calculated. The difference between these two Euclidean distances is hypothesized to serve as a quantitative measure for the similarity between the two corresponding flow fields.

##### Dynamic Time Warping (DTW) Distance

While POD-based Euclidean distances provide a compact measure of structural similarity between flow fields, they neglect temporal information and transient flow features present in in-vivo derived hemodynamics. To complementarily capture dynamic aspects of the flow, the similarity between the temporal POD coefficients ***b***_*i*_*(t)* of the pre/post state and the healthy reference was quantified using Dynamic Time Warping (DTW). DTW is one of the most widely used approaches to assess sequence similarity, first introduced in the context of speech recognition to accommodate temporal distortions and variations in speaking rates [31]. DTW aligns potentially phase-shifted or time-warped dynamics in two time series by locally stretching or compressing the time axis through iteratively defining a warping curve to minimize the cumulative distance between data points. [32] In this study, all ***b***_*i*_*(t)* of the first four modes were normalized for each patient and state. Then, a DTW analysis was applied to each pair of temporal coefficients, e.g. the DTW distance between ***b***_*1*_*(t)* of the post-TAVI state and ***b***_*1*_*(t)* of the healthy state. This resulted in eight DTW distances per patient, defined by the two state-pairs pre-healthy and post-healthy and the four POD modes. Further, in order to compress the four separate DTW distances for the four associated POD modes, a cumulative DTW was determined for every patient. This multivariate DTW analysis was conducted to assess the cumulative change in DTW distance over all four modes, by minimizing the single-mode DTW distances simultaneously while preserving the dependencies between coefficients. The interventional change from pre- to post-state was quantified for the mode-specific and the multivariate DTW distances. A reduction in DTW distance post-TAVI was interpreted as increased similarity of the post-interventional flow to the healthy reference, whereas an increase indicated reduced similarity.

### Data Analysis

After performing the in-vivo and in-silico data processing, three main output quantities were obtained per patient: A scalar composite cardiovascular stress signature, a pre-procedural fHb value, and Euclidean distance change values from the pre- to post-TAVI state in regard to the healthy baseline, calculated either from conventional hemodynamic fields or from POD-based weight values.

The resulting data were then analyzed exploratorily. This included quasi-unsupervised k-means clustering of the point clouds created by the POD-based Euclidean distance ***D***_***Eucl-POD***_ change values and the fHb levels, with the number of clusters defined a priori to k = 2. The resulting cohorts were then compared to the assigned composite cardiovascular stress signatures. Furthermore, a Mann-Whitney U test with a significance threshold of p = 0.05 was conducted to compare the contribution of each mode between the two cohorts.

Finally, to compare POD-based and conventional descriptors, pair-wise k-means analysis (k = 2) were also conducted for combinations of conventional parameters. Their resulting cluster assignments were examined with respect to the composite cardiovascular stress signatures and compared qualitatively to those obtained for the POD-based point clouds.

## Results

### In-vivo Data Results

#### fHb Levels

**Fig. 2:**
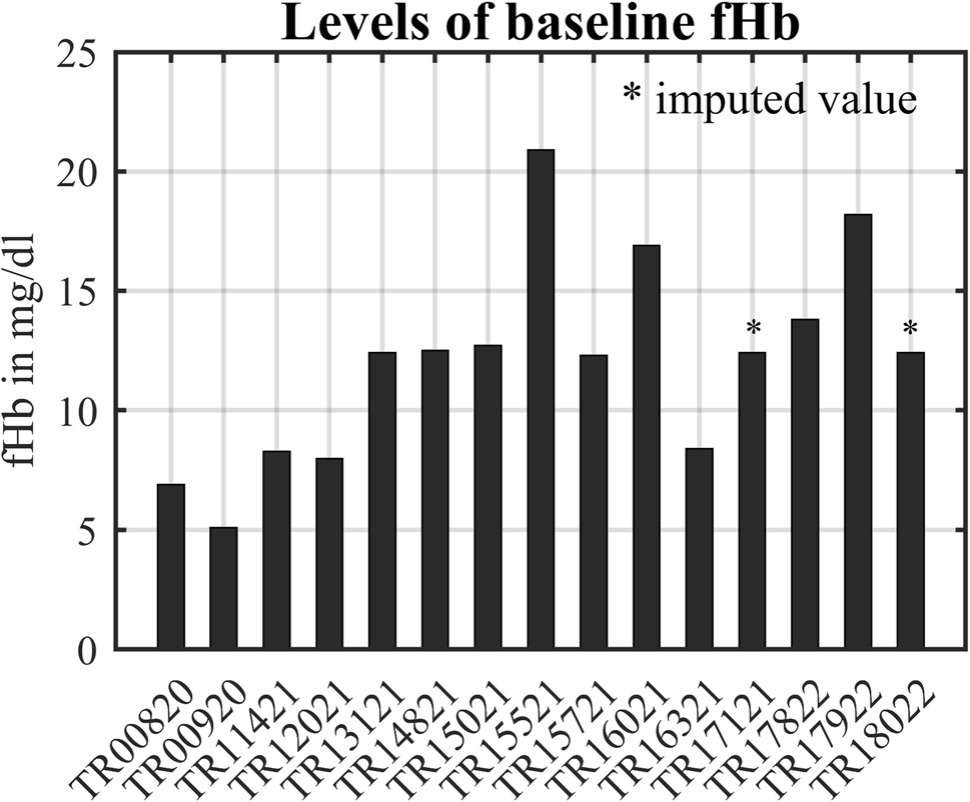
Barplot of clinically measured baseline fHb values. Imputed entries are marked with an asterix

The fHb levels pre-TAVI of the clinical cohort are shown in Fig. 2. Values which were imputed due to missing measurements are marked accordingly.

#### Composite Cardiovascular Stress Signature Determination

**Fig. 3:**
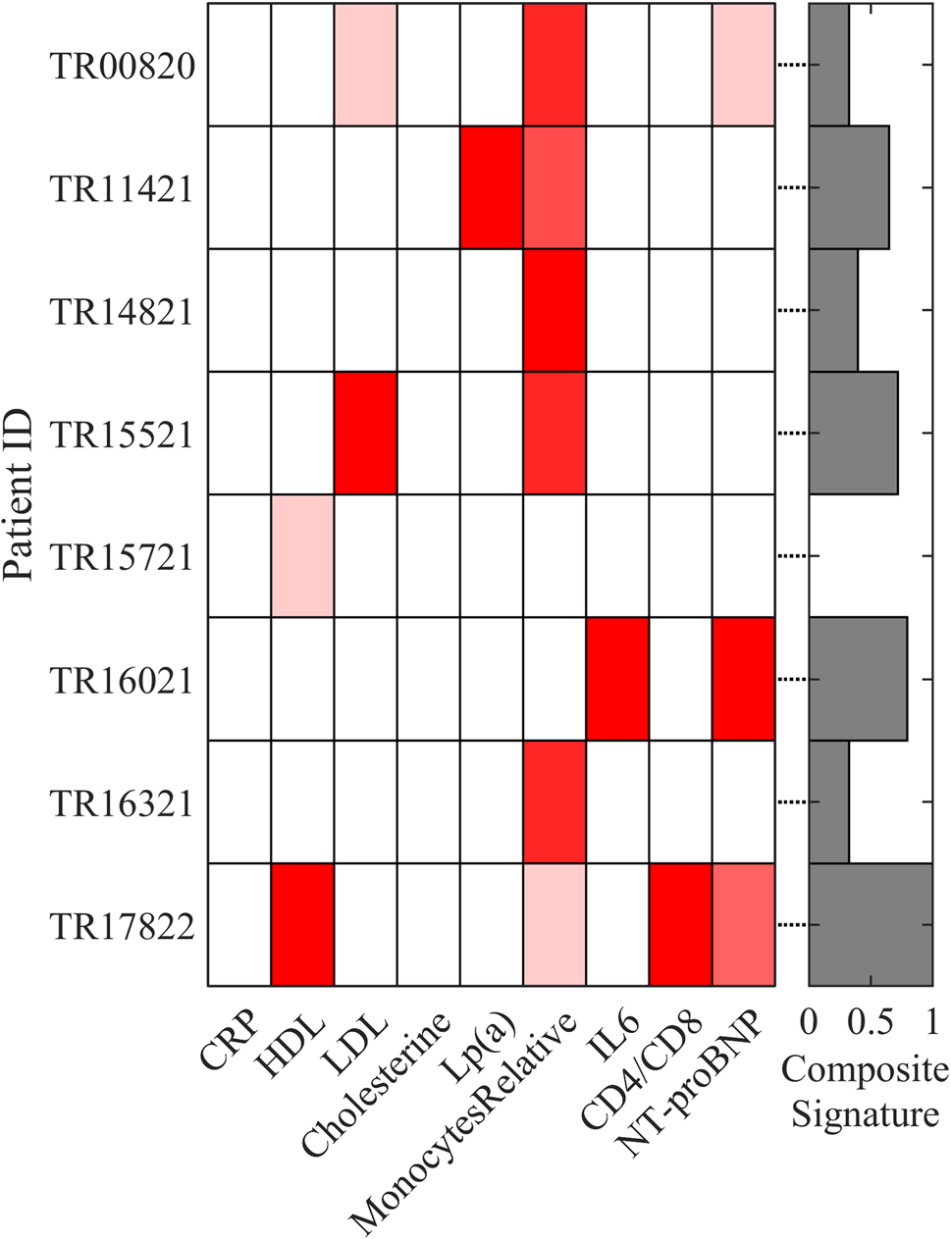
Biomarker value matrix for n = 8 patients, color intensity representing the relative degree of elevation/alteration from physiological range, calculated within the cohort. Right barplot displays the resulting normalized composite cardiovascular stress signature

The biomarker values for the patient cohort are presented in the Supplementary Table 2 (S-III), alongside reference threshold values from established literature. Based on these thresholds, pathologically elevated or altered parameter levels within the patient sub-cohort were identified and the corresponding composite cardiovascular stress signature were subsequently calculated as outlined in the previous chapter. The results are visualized in Fig. 3, where the color intensity of the highlighted matrix cells reflects the relative degree of parameter deviation within the cohort. Greater intensity indicates higher deviation from normative values. The adjacent bar plot displays the final normalized composite cardiovascular stress signature value for each patient, enabling a comparative assessment of individual burden across the study population.

#### Short-Term Outcomes

Of the eight patients, implantation quality was rated as good in four, intermediate in three, and poor in one. Among the intermediate-to-poor cases, three prostheses were positioned too low, with patients TR 114-21 and TR 163-21 subsequently exhibiting aortic insufficiency. The longest hospital stays were observed in patients TR 157-21 (7 days), TR 160-21 (7 days) and TR 178-22 (10 days). Post-procedural delirium occurred in patient TR 160-21, who also developed non-structural bioprosthesis valve dysfunction (BVD), a complication likewise observed in patient TR 178-22.

### In-silico Data Results

After the 8x3 patient-specific, 4D Flow MRI-based CFD models of aortic flow were generated, the hemodynamic fields were evaluated conventionally and using dimensionality reduction techniques.

#### POD: Mode Contours and Coherent Structures

**Fig. 4:**
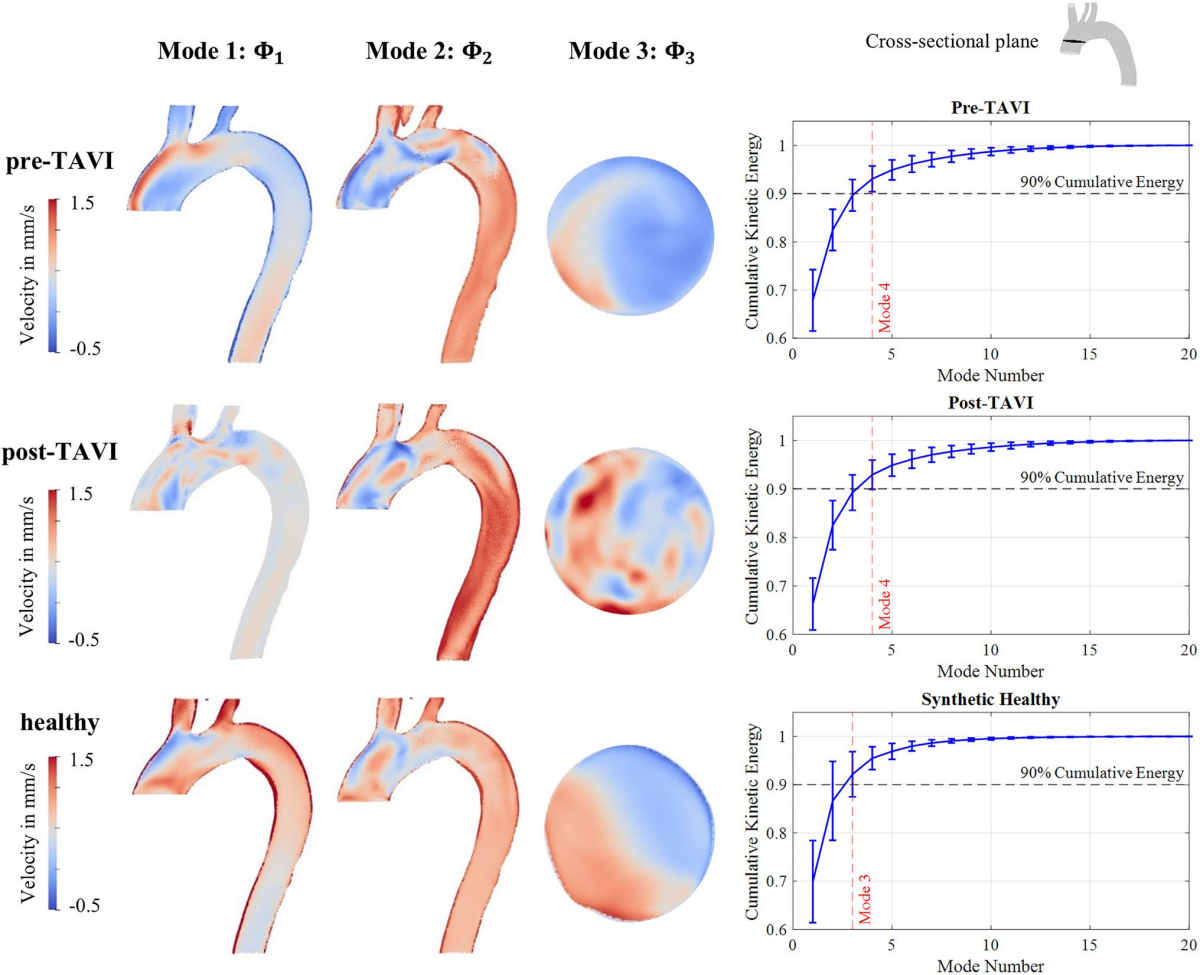
Representative visualization of first three POD mode contours for healthy, pre-TAVI and post-TAVI of TR 008-20. Right column shows the mean mode contributions among all n = 8 subjects, with a threshold marked as horizontal dashed line at 0.9, indicating the cumulative kinetic energy deemed sufficient for flow approximation

Following the application of POD for dimensionality reduction across all n = 8 numerical models, the kinetic energy captured by each mode was evaluated. The cumulative kinetic energy exceeded 90 % within the first three to four modes. Thus, a linear combination of these distinct flow patterns can serve as a reliable approximation of the original flow field. The right panel of Fig. 4 illustrates the cumulative energy distribution as a function of mode number, averaged across the given cohort, for each respective case.

In addition, the extracted POD modes were qualitatively assessed by visualizing their associated flow maps, see Supplementary Material S-IV. These mode contours revealed coherent flow structures across all patients and states and were most clearly identifiable for the healthy case, whereas the pre/post results displayed some degree of variability. Representative contour plots for patient TR 008-20 are visualized in the left panel of Fig. 4. The highest ranked mode predominantly represents the systolic velocity flow originating from the aortic valve. The jet eccentricity and width differed between the different patients and cases. The second mode captures elevated velocities in the descending aorta of the healthy flows, accompanied by lower velocities in the ascending aortic domain. Mode 3 depicts two separate regions exhibiting bidirectional flow, resulting in a cross-sectional bihelical pattern in the healthy ascending aorta.

##### Reconstruction of Original Flow Field

The original flow was reconstructed through a linear combination of the POD modes for each instantaneous snapshot. This procedure yielded 20 temporal POD coefficients, which are shown in the Supplementary Material S-V for the first four modes. The accuracy of the reconstruction improved with the number of modes included for the reconstruction. Overall satisfactory reconstruction was obtained using the first four modes, which captured over 90 % of total kinetic energy, with R^2^ ≈ 0.85 (median RMSE ≈ 0.04, median absolute deviation ≈ 0.02 m/s, median relative error ≈ 21 %). The corresponding distributions are illustrated as boxplots in Supplementary Material S-VI.

#### POD: Dynamic Time Warping Distance

**Fig. 5:**
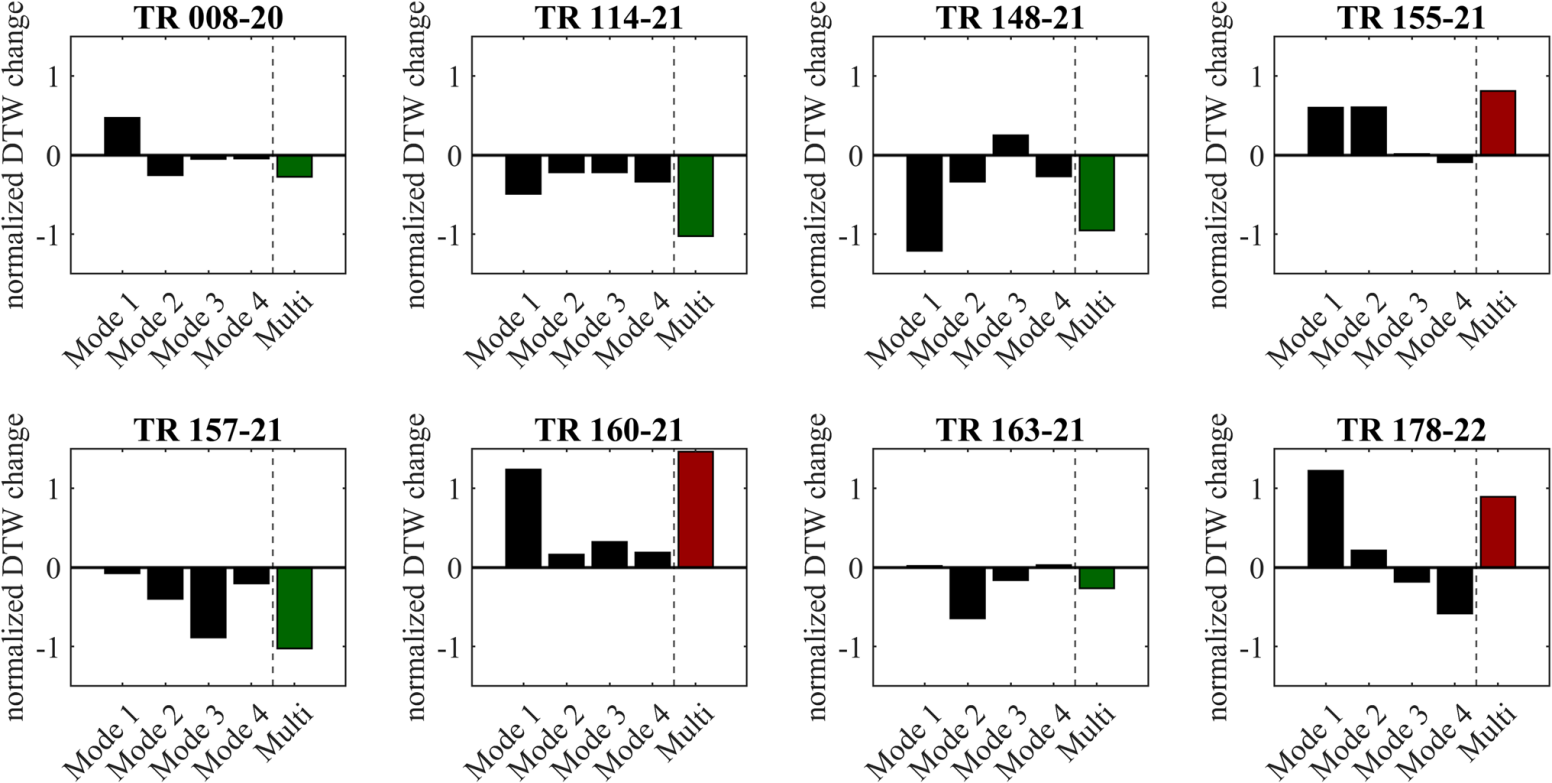
Barplots of the changes in dynamic time warping distance from pre- to post-state to healthy reference. Black bars represent the mode-specific DTW changes, while the colored bars quantify the multivariate DTW results. Green bar indicates improved sequence similarity, red indicates decreased similarity

As illustrated in Fig. 5, the multivariate DTW distance increased in TR 155-21, TR 160-21 and TR 178-22, while it decreased in the remaining five patients. For single-mode DTW results, distance changes varied across patients and states. Nonetheless, in the three patients of increased multivariate DTW distance, most individual DTW distances also increased, whereas they predominantly decreased in the other patients.

#### POD: Euclidean Distances and Flow Restoration Metric

**Fig. 6:**
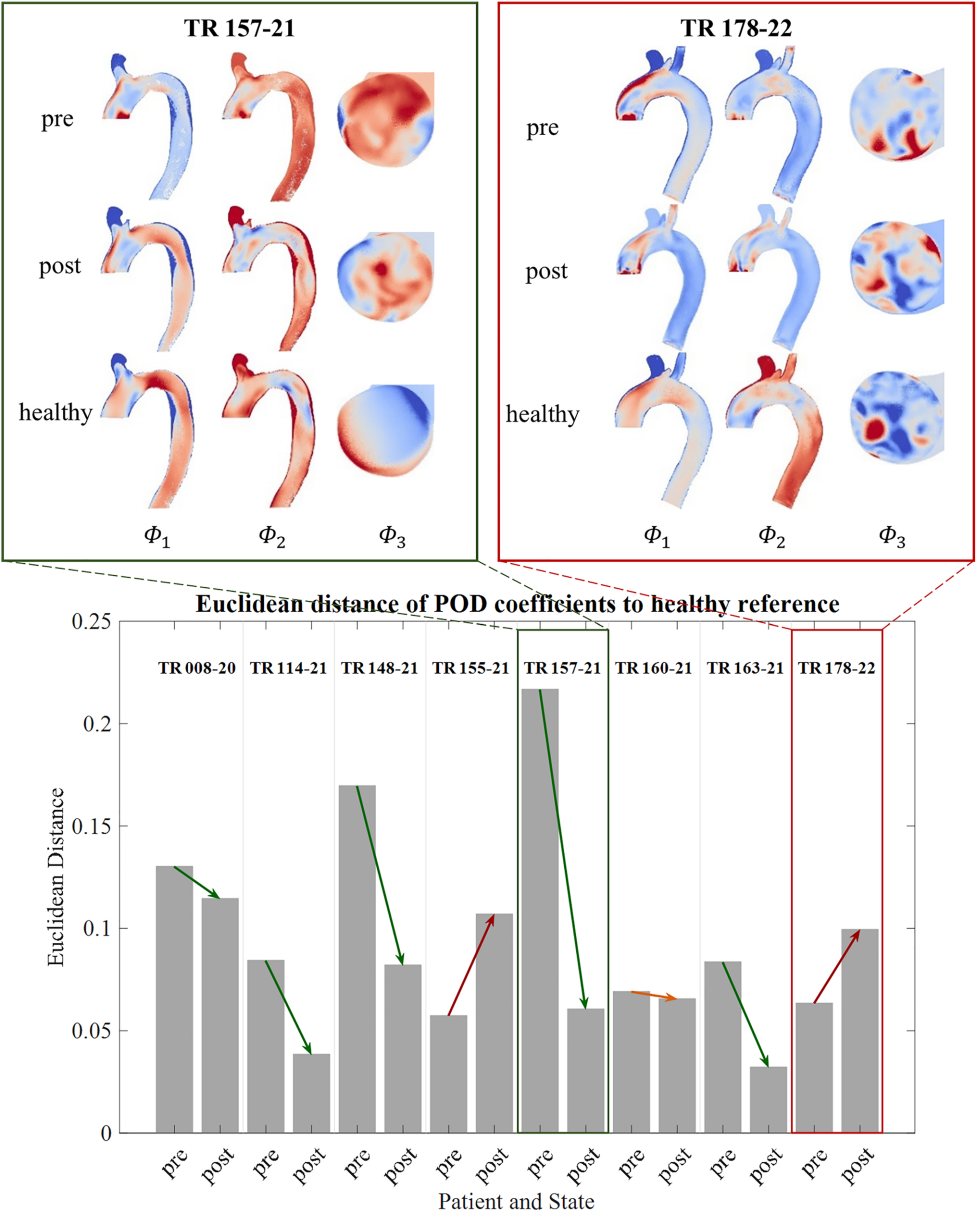
Barplot of the changes in Euclidean distance D_Eucl-POD_ for the n = 8 patients. Decrease in distance is marked by green arrow, indicating better flow restoration, increase in distance is marked by red arrow, indicating worse restoration. Two patient-specific POD contours are shown in upper panels: TR 157-21 as a representation of the better restoration group, TR 178-22 as a representative of the worse restoration group

Then, the Euclidean distances ***D***_***Eucl-POD***_ between the time-averaged POD coefficient vectors ***B*** of the pre- and post-TAVI state and the healthy baseline were computed and compared. These results are summarized in Fig. 6. A reduction in ***D***_***Eucl-POD***_ following the intervention indicates a shift toward the healthy flow pattern, suggesting partial flow restoration. This trend was observable in five out of eight patients in the numerical cohort. Conversely, an increase in distance reflects a further deviation from the healthy reference state, which was the case for the patients TR 155-21, TR 160-21 and TR 178-22, thus matching the patients with increased DTW distances.

Additionally, representative mode contour plots are presented for a higher and lower degree of flow restoration. As a representative example of the high-restoration group, the first two mode contours of subject TR 157-21 demonstrate a strong resemblance between the post-state and the healthy baseline. A pronounced transformation in flow structure is evident from the pre- to the post-state. Specifically, the first pre-TAVI modes are characterized by overall low velocity in the aortic domain with a local, supravalvular peak. In contrast, the corresponding post-TAVI mode reveals more uniformly distributed flow velocities in the descending aorta with a flow jet in the ascending section. Additionally, the third post-TAVI mode reveals a more pronounced bi-regional distribution of flow, whereas the corresponding pre-TAVI mode displays predominantly unidirectional flow across the cross-section. In contrast, the first two modes of subject TR 178-22 exhibit no discernible differences between the pre- and post-state but pronounced alterations to the healthy reference mode contours.

### Data Analysis Results: In-vivo and In-silico

#### K-means Clustering

**Fig. 7:**
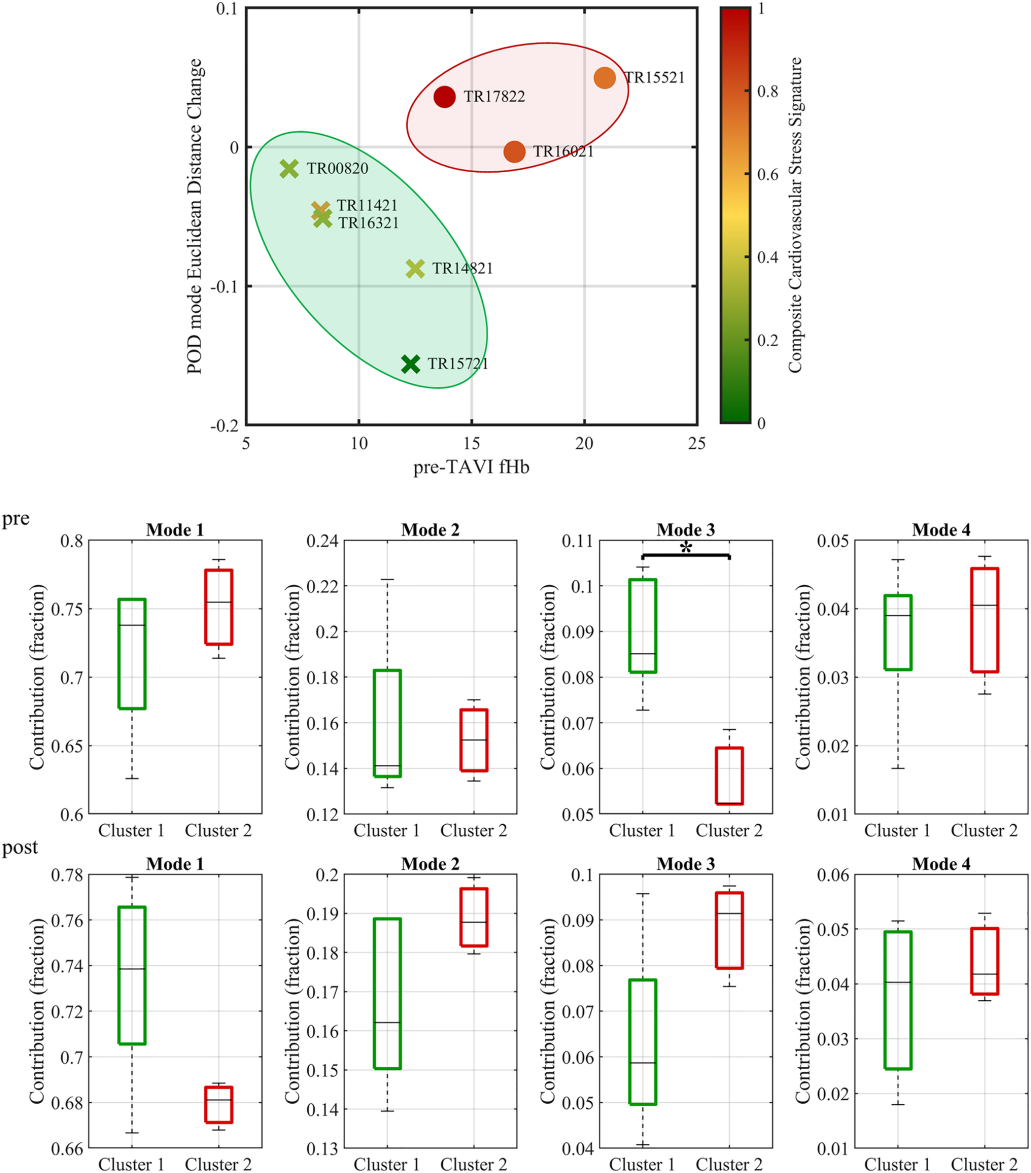
K-means clustering of point cloud created by pre-TAVI fHb levels and Euclidean distance change **D**_**Eucl-POD**_ from pre- to post-TAVI state. Upper panel: Scatter plot, with coloring based on individual cardiovascular stress signature, resulting in two distinct clusters, colored according to the dominant color of their respective members. B) Boxplots of individual mode contributions of the first four POD modes, categorized for the two clusters (green and red) and the two flow states (pre and post). Significant differences (p < 0.05) are present for third mode in the pre-TAVI state

Quasi-unsupervised k-means clustering was applied to the point cloud constructed by the pre-TAVI fHb values and the corresponding changes in Euclidean distance of POD mode coefficients (***D***_***Eucl-POD***_). This analysis yielded two distinct clusters, as illustrated in the upper panel of Fig. 7. The identified clusters further showed correspondence to the cardiovascular stress signature distribution. Patients with more pathological cardiovascular stress (value close to 1) exhibit higher pre-TAVI fHb levels and larger ***D***_***Eucl-POD***_ values, indicating limited post-interventional flow restoration. In contrast, the group with more physiological cardiovascular stress (value close to 0) is characterized by lower pre-TAVI fHb and smaller ***D***_***Eucl-POD***_, suggesting a more physiological post-TAVI flow state. To further examine the flow characteristics of each cluster, the average modal contributions before and after TAVI were evaluated cluster-wise. While notable differences can be observed for the post-procedural contribution of modes 1 through 3, the most obvious distinction emerges in the pre-TAVI contribution of the third mode. Specifically, patients in Cluster 2 (high-risk) demonstrate much lower mode 3 contributions, implying a disruption or suppression of the corresponding vortex structure within the ascending aortic flow due to AS.

#### Comparison of Conventional Parameters and POD

**Fig. 8:**
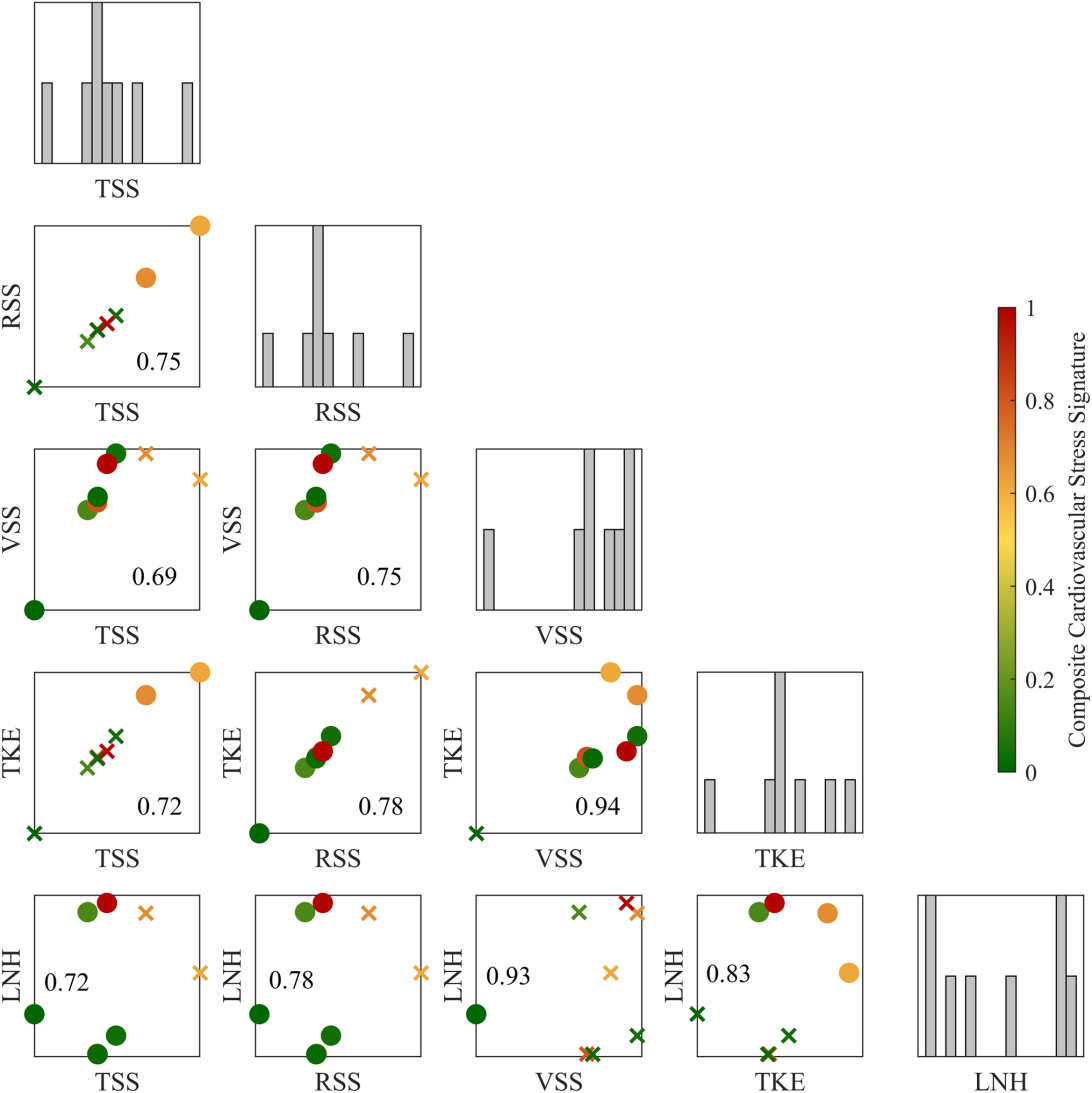
Pairwise k-means clustering of conventional parameters with silhouette score as metric of cluster separability and scatter-point coloring based on individual composite cardiovascular stress signatures. TSS: Total Shear Stress, RSS: Reynolds Shear Stress, VSS: Viscous Shear Stress, TKE: Turbulence Kinetic Energy, LNH: Local Normalized Helicity

The mean Euclidean distances (***D***_***Eucl-conv***_) were computed for the conventional parameters TSS, VSS, RSS, TKE and LNH, between both the pre- and post-interventional state and the healthy reference model. Pairwise combinations of conventional parameters were explored using k-means clustering (k = 2) and compared to the clustering obtained from the POD-based point cloud. As shown in Fig. 8, several combinations of conventional parameters, particularly including TKE, VSS and LNH., produced visually well-separated clusters in feature space. This suggests that data points are well grouped within their respective clusters and are distinctly separated from neighboring clusters. However, these clusters did not align consistently with the composite cardiovascular stress signatures of the patients.

## Discussion

Alterations in aortic flow patterns are known to reflect cardiovascular health and restoration of normal hemodynamic profiles has been proposed as a therapeutic target in AS management. However, quantification of conventional hemodynamic markers is typically constrained to discrete temporal and spatial analyses, preventing holistic capture of the full four-dimensional flow field. As a result, only a fraction of the information embedded within hemodynamic structures is utilized, limiting the potential to fully define physiological flow patterns and their pathological alterations, as well as post-procedural restoration. To address this limitation, we presented a novel, unsupervised approach for comprehensive characterization and comparison of patient-specific aortic hemodynamics before and after TAVI relative to healthy baselines. We further contextualize these flow structures and their restoration with clinical parameters related to adverse TAVI outcome and cardiovascular health.

Accordingly, this study yielded three key outcomes that will be detailed in the subsequent sections:

1. Coherent flow structures of four-dimensional aortic flows were identified using POD.
2. Flow restoration degrees post-TAVI was quantified using POD-based similarity metrics, unsupervised clustering and associated composite cardiovascular stress signature.
3. POD-based markers yielded clinically interpretable clusters associated with established biochemical parameters and short-term outcomes.

### POD-Based Evaluation of Coherent Flow Structures and Holistic Similarity Metrics

In this study, the first three to four modes accounted for over 90 % of the system’s total energy, providing an accurate reconstruction of the underlying dynamics with these structures with R^2^ ≈ 0.9. As shown in Supplement S-IV, the leading modes share consistent resemblance across the subject cohort (Mode 1: main systolic velocity jet, Mode 2: higher downstream velocity in descending aorta, Mode 3: bi-directional flow in ascending aortic cross-section) yet exhibit quantitative differences. Accordingly, this study proposes the use of their temporal POD coefficients ***b(t)*** and the time-averaged coefficient vectors ***B*** instead, which quantify each mode’s relative energy content level. Moreover, the Euclidean distance between the coefficient vectors, and the multivariate DTW distance between the temporal coefficients provide a quantitative similarity metric. These two distances quantify complementary aspects of flow-field similarity. While Euclidean POD distance reflects structural similarity, DTW evaluates time-resolved evolution of these structures and can capture phase shifts or temporal distortions undetected by static weight comparisons. Considering both metrics therefore provides a more complete assessment: the time-averaged Euclidean distance indicates whether the modal composition approaches the healthy state, while DTW shows whether its temporal dynamics also converge. Improvements in both measures suggest simultaneous recovery of structure and dynamics. In this study, this distance was used to assess flow restoration after TAVI by comparison of the pre- and post-procedural coefficient vectors to a healthy baseline. Both the comparison using ***b(t)*** and ***B*** have identified identical patient groupings. In 5 of the 8 patients in the sub-cohort, the DTW distance decreased after the intervention, indicating improved temporal restoration of flow. For the Euclidean distance, 5 of the 8 patients reflected improved structural restoration, and one borderline case with almost no change in flow similarity.

### Flow Restoration and Association with Composite Cardiovascular Stress Signature

To systematically evaluate the discriminative potential of POD, k-means clustering was performed using POD-derived metrics in combination with pre-TAVI fHb levels, as shown in Fig. 7. Two distinct clusters emerged: one characterized by higher pre-TAVI fHb levels and predominantly positive Euclidean distance changes (indicating lower similarity to healthy flow), another with lower pre-TAVI fHb levels and negative Euclidean distance changes (indicating greater similarity to healthy flow). The first cluster corresponded to more pathological composite cardiovascular stress signatures, while the second was generally associated with more physiological signatures. The POD-based analysis further enabled the examination of mode-specific contributions between these clusters and across patient states. The difference in mode 3 between the two clusters can be interpreted as an absence of bidirectional (bi-helical) flow in the high stress signature cluster. This aligns with studies on helical flow, for instance conducted by Morbiducci et al., which emphasize the physiological relevance of a bihelical structure in the ascending aorta [4, 27]. The first mode experiences a decline in energy contribution comparing pre- and post-state for the high stress signature cluster, suggesting that preserving the energy of the first and most dominant pattern (> 70 %) may be beneficial for favorable outcome.

The clinical documentation, presented in Supplementary Material S-I, provides further support for clinical validity of the clustering results. Patients TR 160-21 and TR 178-22, who exhibited the highest NT-proBNP levels and the most pathological biomarker values, experienced post-procedural complications due to bioprosthetic valve dysfunction. Further, both procedures required prolonged hospital stays (7 and 10 days, respectively), with TR 160-21 additionally developing post-procedural delirium. Importantly, these adverse outcomes did not correlate with standard baseline risk stratification tools (e.g., NYHA classification, EuroScore, STS Score, etc.) nor with measures of prosthesis positioning quality. For instance, patients TR 148-21, TR 155-21, TR 157-21 and TR 163-21 were rated as having suboptimal prosthesis positioning but did not experience any clinically relevant complications during follow-up, whereas TR 160-21 and TR 178-22, despite technically adequate prosthesis placement, developed significant post-procedural complications. Further, the risk scores of each patient were not consistent across different stratifications, underlining the limitation of these tools.

This discrepancy emphasizes the potential relevance of the degree of flow restoration as a complementary descriptor of procedural outcome in the given cohort. Patients with poor restoration of flow dynamics, mirrored in more pathological stress signatures, were more likely to experience complications, whereas others with procedural imperfections but more physiological flow restoration fared comparatively well. Thus, the degree of hemodynamic recovery appears to be a central decisive factor for prognosis in our cohort, which should not be excluded in the overall assessment of procedural success. Overall, the clustering results highlight the strong discriminative capacity of the POD-derived metrics, consistent with known clinical outcomes. This aligns with findings by Borja et al., who used POD to differentiate ventricular flow patterns among healthy subjects and patients with dilated and hypertrophic cardiomyopathy [21].

However, given the limited cohort size, this observation should be interpreted as exploratory rather than predictive. A central limitation of this study is the overall small final sub-cohort which resulted from strict technical inclusion criteria regarding imaging coverage, image quality and availability of biochemical measurements. Therefore, the present work primarily aimed to develop and evaluate a novel flow analysis framework, rather than to carry out a statistically powered clinical study. Although the paired pre-/post-TAVI design enables within-subject comparison, validation in a larger and more heterogeneous cohort will be necessary to investigate generalizability.

### Comparison of Conventional and POD-Based Flow Markers

Compared with conventional hemodynamic parameters, POD-derived metrics demonstrated stronger clinical interpretability during analysis. This underlines the advantage of extracting coherent, physically realistic flow structures that align with the effects of biological processes, as opposed to probe-wise assessment of derived conventional fields. It enables the identification of the actual flow structures leading to the separation into two groups and simplifies the clinical interpretation of the results. Supporting this, the pairwise clustering in Fig. 8 reveals high discriminative power for the combined assessment of, for instance, LNH and TKE. However, the resulting clusters do not correspond to clinically meaningful groupings, as they contain a mixture of patients with both high and low clinical burden. Moreover, the hemodynamic phenomenon behind the differences in LNH or TKE values remains unknown. Thus, while conventional parameters provide mathematical separability, they do not yield clinically interpretable clusters to the same extent as the POD-based descriptors This further emphasizes the added relevance of holistic, dimensionality reduction-based flow analysis tools.

Moreover, these POD tools can also be directly applied to in-vivo velocity data and do not require the calculation of gradient or curl fields, which would lead to additional, propagated uncertainties. Though in-vivo data is inherently noisy as well and come with their own challenges such as low-resolution, techniques such as robust POD or data assimilation allow to consider for these limitations and have been applied for various tasks in fluid mechanics [33, 34].

The clinical evaluation of these study results was derived from 30 days follow-up data and biochemical associations, drawing on established relationships between adverse events and specific biomarkers reported in previous clinical studies. The reliability of this approach therefore depends on accurate laboratory analyses, the predictive validity of these biomarkers, and the assumption that 30-days follow-up data are representative of long-term outcomes. Nevertheless, these are considered reasonable surrogates for durable outcomes to demonstrate the overall applicability of the POD-based method and the relevance of quantifying post-interventional flow restoration. Future work should validate these findings in a larger cohort with longitudinal outcome data and apply this method on in-vivo 4D Flow MRI flow velocity directly to eliminate numerical uncertainties.

In summary, two POD-based flow analysis metrics were developed to quantify the degree of aortic flow restoration following TAVI by assessing their structural and dynamical similarity to healthy hemodynamics. The underlying dimensionality reduction method provides a holistic and interpretable representation of complex flows. Subsequent clustering exhibited strong discriminative potential between different levels of restoration and significant associations with composite cardiovascular stress signatures, offering a more nuanced framework for identifying vulnerable subgroups aligning with known clinical outcomes. Following validation in a larger population, this approach could inform the design of next-generation TAVI devices optimized for flow restoration. Furthermore, integrating holistic morphological descriptors with POD-derived hemodynamic markers into population-based reduced-order models could aid the design of fast, clinically applicable diagnostic tools and therefore enhance translation into clinical practice.

## Supplementary Information

Below is the link to the electronic supplementary material.

Supplementary file1 (DOCX 2478 KB)

## Data Availability

A template MATLAB script is available on Zenodo: 10.5281/zenodo.17063639
