# Supplementary material for: Development of a Novel Holistic Assessment Strategy of TAVI-Induced Flow Restoration via Dimensionality Reduction Techniques
Source: Cardiovasc Eng Technol. 2026 May 14;17(4):439–56. doi: 10.1007/s13239-026-00840-2 (PMC13442565; doi:10.1007/s13239-026-00840-2)
Supplement: Supplementary file 1 [file 13239_2026_840_MOESM1_ESM.docx]

**SUPPLEMENTARY MATERIAL**

**Development of a novel holistic assessment strategy of TAVI-induced flow restoration via dimensionality reduction techniques**

Tianai Wang^1^, Tobias Zeus^2^, Christine Quast^2^, Ayse Sedef Ceylan^2^, Kathrin Klein^2^, Malte Kelm^2,3^, Francesca Dell’Agnello^4^, Simona Celi^4^, Ulrich Steinseifer^1^, Michael Neidlin^1^

**Affiliations:**

1: Department of Cardiovascular Engineering, Institute of Applied Medical Engineering, Medical Faculty, RWTH Aachen University, Aachen, Germany

2: Department of Cardiology, Pulmonary Diseases and Vascular Medicine, Heinrich-Heine University, Düsseldorf, Germany

3: CARID, Cardiovascular Research Institute Düsseldorf, Heinrich-Heine University, Düsseldorf, Germany

4: BioCardioLab, Bioengineering Unit, Fondazione Monasterio, Massa, Italy

***Correspondence:**

Name: Tianai Wang

Address: Department of Cardiovascular Engineering, Institute of Applied Medical Engineering, Forckenbeckstr. 55, 52074 Aachen, Germany

Email address: wang@ame.rwth-aachen.de

Phone number: +49 241 80-47434

ORCID-ID: 0009-0000-5851-3915

**S-I: Clinical characteristics and procedural outcomes of sub-cohort**

Supplementary Table 1a and 1b summarize key clinical, pre-, inter- and post-procedural (30 days) characteristics, i.e. surgical risk assessments (Logistic EuroScore, Standard EuroScore, EuroScore II, STS Score), thromboembolic risk (CHA2DS2-VASc score), prosthesis implantation quality, procedure duration, hospital stay and post-procedural complications. Notably, adverse post-TAVI events, such as valve dysfunction or post-procedural delirium, are not consistently associated with baseline risk scores or prosthesis quality.

**Supplementary Table 1a** Surgical risk assessment scores pre-TAVI.

|  | **EuroScore Logistic** | **EuroScore Standard** | **EuroScore II** | **STS Score** | **CHA2DS2-VASc Score** |
| --- | --- | --- | --- | --- | --- |
| **TR 008-20** | 16.67 | 10 | 2.11 | 1.837 | 4 |
| **TR 114-21** | 3.51 | 5 | 1.06 | 0.884 | 4 |
| **TR 148-21** | 14.41 | 10 | 5.1 | 2.448 | 5 |
| **TR 155-21** | 15.42 | 9 | 3.4 | 1.664 | 4 |
| **TR 157-21** | 17.9 | 10 | 10.53 | 1.456 | 4 |
| **TR 160-21** | 19.94 | 10 | 2.61 | 10.531 | 7 |
| **TR 163-21** | 18.25 | 10 | 2.38 | 2.424 | 4 |
| **TR 178-22** | 7.48 | 7 | 1.79 | 10.041 | 5 |

**Supplementary Table 1b** Procedural and post-procedural (30 days post-procedure) characteristics and complications. AI I° and AI II° refer to aortic insufficiency grade I and II.

|  | **Prosthesis implantation** | **Prosthesis evaluation** | **Inter-vention duration** | **Hospital stay** | **Post-TAVI complication** | **Bioprosthesis valve dysfunction** |
| --- | --- | --- | --- | --- | --- | --- |
|  | 1: good  2: medium  3: bad |  | minutes | days |  | 0: none  1: Structural deterioration  2: non-structural dysfunction  3: thrombosis  4: endocarditis |
| **TR 008-20** | 1 | - | 50 | 4 | - | 0 |
| **TR 114-21** | 3 | AI II° | N/A | 5 | - | 0 |
| **TR 148-21** | 2 | too low | N/A | 6 | - | 0 |
| **TR 155-21** | 2 | too low | 63 | 6 | - | 0 |
| **TR 157-21** | 1 | - | 53 | 7 | - | 0 |
| **TR 160-21** | 1 | - | 77 | 7 | delirium | 2 |
| **TR 163-21** | 2 | borderline low, AI I° | 105 | 6 | - | 0 |
| **TR 178-22** | 1 | - | 147 | 10 | - | 2 |

**S-II: Hemodynamic Parameters**

*Viscous Shear Stress*

Viscous shear stresses (VSS) arise from molecular diffusion in both laminar and turbulent flow. They are associated with the strain rate governing the deformation of fluid elements and can be expressed as the following stress tensor:

$$T=\left( \begin{matrix} 2\mu\frac{\partial u}{\partial x} & \mu(\frac{\partial v}{\partial x}+\frac{\partial u}{\partial y}) & \mu(\frac{\partial w}{\partial x}+\frac{\partial u}{\partial z}) \\ \mu(\frac{\partial v}{\partial x}+\frac{\partial u}{\partial y}) & 2\mu\frac{\partial v}{\partial y} & \mu(\frac{\partial w}{\partial y}+\frac{\partial v}{\partial z}) \\ \mu(\frac{\partial w}{\partial x}+\frac{\partial u}{\partial z}) & \mu(\frac{\partial w}{\partial y}+\frac{\partial v}{\partial z}) & 2\mu\frac{\partial w}{\partial z} \end{matrix} \right)=\left( \begin{matrix} \sigma_{xx} & \tau_{xy} & \tau_{xz} \\ \tau_{yx} & \sigma_{yy} & \tau_{yz} \\ \tau_{zx} & \tau_{zy} & \sigma_{zz} \end{matrix} \right)$$

(1)

Here, the diagonal entries *σ* represent the normal stresses acting on the fluid elements and the off-diagonal terms *τ* describe the shear stresses. *μ* denotes the fluid dynamic viscosity and the variables *u*, *v*, and *w* describe the velocity components in *x*, *y* and *z*-direction, respectively, in laminar flows or their statistically averaged quantities in turbulent flows. The VSS tensor components can be given in index notation as follows:

$$\tau_{ij, viscous}=\mu\left( \frac{\partial u_{i}}{\partial x_{j}}+\frac{\partial u_{j}}{\partial x_{i}} \right)=2{\mu D}_{ij}$$

(2)

𝐷_𝑖𝑗_ is the strain rate tensor and 𝑢_𝑖_ defines the *i*-th component of the velocity (instantaneous for laminar flows, time-averaged for turbulent flow). For practical purposes, it is often advantageous to define a scalar equivalent stress, for instance [1]:

$$\tau_{viscous}=\frac{1}{\sqrt{3}}\sqrt{{\sigma_{xx}}^{2}+{\sigma_{yy}}^{2}+{\sigma_{zz}}^{2}-{(\sigma}_{xx}\sigma_{yy}+\sigma_{xx}\sigma_{zz}+\sigma_{yy}\sigma_{zz)}+3({\tau_{xy}}^{2}+{\tau_{xz}}^{2}+{\tau_{yz}}^{2})}$$

(3)

*Reynolds Shear Stress*

Using the Boussinesq’s assumption, the Reynolds stress is given by Equation (4) in index notation [1,2]:

$$\tau_{ij, Reynolds}=-\rho\overset{⃐}{{u^{'}}_{i}{u^{'}}_{j}}=\mu_{t}\left( \frac{\partial u_{i}}{\partial x_{j}}+\frac{\partial u_{j}}{\partial x_{i}} \right)-\frac{2}{3}\rho k\delta_{ij}$$

(4)

with 𝜇_𝑡_ being the eddy viscosity, 𝜌 the density, 𝑘 the turbulence kinetic energy and 𝛿_𝑖𝑗_ the Kronecker delta. The eddy viscosity 𝜇_𝑡_ depends on the turbulence model, e.g., determined by the turbulent kinetic energy *k* and the turbulent frequency ω for the Shear Stress Transport model.

*Total Shear Stress*

The total shear stress (TSS) tensor combines the VSS and RSS contribution and is given in index notation:

$$\tau_{ij,total}=\tau_{ij, viscous}+\tau_{ij, Reynolds}=\mu\left( \frac{\partial u_{i}}{\partial x_{j}}+\frac{\partial u_{j}}{\partial x_{i}} \right)+\mu_{t}\left( \frac{\partial u_{i}}{\partial x_{j}}+\frac{\partial u_{j}}{\partial x_{i}} \right)-\frac{2}{3}\rho k\delta_{ij}=(\mu+\mu_{t})\left( \frac{\partial u_{i}}{\partial x_{j}}+\frac{\partial u_{j}}{\partial x_{i}} \right)-\frac{2}{3}\rho k\delta_{ij}$$

(5)

The corresponding scalar equivalent stress ultimately equals to Equation (6):

$$\tau_{total}=\frac{1}{\sqrt{3}}\cdot\ldots$$

$$\ldots\sqrt{{\sigma_{xx,t}}^{2}+{\sigma_{yy,t}}^{2}+{\sigma_{zz,t}}^{2}-{(\sigma}_{xx,t}\sigma_{yy,t}+\sigma_{xx,t}\sigma_{zz,t}+\sigma_{yy,t}\sigma_{zz)}+3({\tau_{xy,t}}^{2}+{\tau_{xz,t}}^{2}+{\tau_{yz,t}}^{2})}$$

(6)

Where 𝜎_𝑖𝑗,t_ denotes the total stress components 𝜎_𝑖𝑗,total_.

*Turbulent Kinetic Energy*

The turbulent kinetic energy (TKE) quantifies the kinetic energy associated with velocity fluctuations $u_{i}'$ and can be computed from the Reynolds normal stresses:

$$TKE=\frac{1}{2}\overset{⃐}{{u^{'}}_{i}{u^{'}}_{i}}=\frac{1}{2}(\overline{({u')}^{2}+({v')}^{2}+({w')}^{2}})$$

(7)

*Helicity*

Helicity H describes the helical motion in three-dimensional flow and is defined as the scalar product of the velocity $\boldsymbol{V}(\boldsymbol{x};t)$ and vorticity field $\omega\left( \boldsymbol{x};t \right)= \nabla\times\boldsymbol{V}(\boldsymbol{x};t)$:

$$H(\boldsymbol{x},t)=\boldsymbol{V}(\boldsymbol{x};t)\cdot\omega(\boldsymbol{x};t)$$

(8)

The local normalized helicity LNH serves as the corresponding normalized quantity [3]:

$$LNH\left( \boldsymbol{x};t \right)= \frac{\boldsymbol{V}(\boldsymbol{x};t)\cdot\omega(\boldsymbol{x};t)}{\left| \boldsymbol{V}\left( \boldsymbol{x};t \right) \right||\omega\left( \boldsymbol{x};t \right)|}, -1\leq LNH\leq1$$

(9)

**S-III: Biomarker values from clinical dataset and physiological ranges**

Supplementary Table 2 summarizes the measured values of the biomarkers of interest for the clinical cohort (n = 15). The measurements were taken at University Hospital Düsseldorf as baseline values pre-TAVI. One should note that healthy reference ranges were taken from existing clinical studies, thus, certain differences in threshold values may exist between studies.

**Supplementary Table 2** Biomarkers of interest, measured values and physiological range. Imputed values are marked with an asterisk. Values outside of physiological range are highlighted in bold.

| **Marker** | **CRP** | **HDL** | **LDL** | **Total Cholest.** | **Lp(a)** | **Monocy. counts** | **IL-6** | **CD_4_/**  **CD_8_** | **NT-proBNP** |
| --- | --- | --- | --- | --- | --- | --- | --- | --- | --- |
| **Unit** | **mg/dl** | **mg/dl** | **mg/dl** | **mg/dl** | **nmol/l** | **%** | **pg/ml** | **-** | **pg/ml** |
| **TR**  **008-20** | 0.1 | 85 | **114** | 195 | 41* | **13*** | 5* | 2.8 | **1783** |
| **TR**  **114-21** | 0.1 | 60 | 65 | 133 | **227** | **8.6** | 7 | 2.7* | 198 |
| **TR**  **148-21** | 0.3 | 48 | 77 | 130 | 23 | **8.9** | 3 | 2.5 | 261 |
| **TR**  **155-21** | 0.1 | 51 | **126** | 199 | 55 | **12*** | 4* | 2.9* | 347 |
| **TR**  **157-21** | 0.1 | **39** | 64 | 114 | 10 | 6.5 | 2* | **5.6** | 170 |
| **TR**  **160-21** | 0.4 | 54 | 41 | 108 | 8 | 5.6 | **14** | 2.5 | **2346** |
| **TR**  **163-21** | 0.3 | 48 | 67 | 128 | 32* | 6.6* | 2* | 2.9* | 199 |
| **TR**  **178-22** | 0.1 | **30** | 98 | 167 | 47* | 8.1 | 4* | **0.8** | **1891** |
| **Physiol. Range** | **< 0.8** ^[4]^ | **> 40** ^[5]^ | **< 100** ^[5]^ | **< 200** ^[6]^ | **< 120** ^[7]^ | **< 8** ^[8]^ | **< 7** ^[9]^ | **1-3** ^[8]^ | **< 400** ^[10]^ |

**S-IV: POD mode contours of patients**

Supplementary Table 3 presents the POD contour plots of all n = 8 patients, which describe the percentage variation from the mean velocity field in the pre-TAVI, post-TAVI and healthy state. They correspond to the proportion of total flow variance, i.e. coherent flow structures, captured by each mode. The contour plots provide a spatial representation of the modal structures, allowing qualitative comparison of flow patterns.

**Supplementary Table 3** Velocity contour plots of POD modes 1-3 for each patient in pre-TAVI, post-TAVI and healthy state, representing percentage variation from the mean velocity field.

| **TR 008-20** | Mode 1: $\Phi_{1}$ | Mode 2: $\Phi_{2}$ | Mode 3: $\Phi_{3}$ |
| --- | --- | --- | --- |
| Pre-TAVI   |  |  |  |
| Post-TAVI   |  |  |  |
| Healthy   |  |  |  |
| **TR 114-21** | Mode 1: $\Phi_{1}$ | Mode 2: $\Phi_{2}$ | Mode 3: $\Phi_{3}$ |
| Pre-TAVI   |  |  |  |
| Post-TAVI   |  |  |  |
| Healthy   |  |  |  |

| **TR 148-21** | Mode 1: $\Phi_{1}$ | Mode 2: $\Phi_{2}$ | Mode 3: $\Phi_{3}$ |
| --- | --- | --- | --- |
| Pre-TAVI   |  |  |  |
| Post-TAVI   |  |  |  |
| Healthy   |  |  |  |
| **TR 155-21** | Mode 1: $\Phi_{1}$ | Mode 2: $\Phi_{2}$ | Mode 3: $\Phi_{3}$ |
| Pre-TAVI   |  |  |  |
| Post-TAVI   |  |  |  |
| Healthy   |  |  |  |

| **TR 157-21** | Mode 1: $\Phi_{1}$ | Mode 2: $\Phi_{2}$ | Mode 3: $\Phi_{3}$ |
| --- | --- | --- | --- |
| Pre-TAVI   |  |  |  |
| Post-TAVI   |  |  |  |
| Healthy   |  |  |  |
| **TR 160-21** | Mode 1: $\Phi_{1}$ | Mode 2: $\Phi_{2}$ | Mode 3: $\Phi_{3}$ |
| Pre-TAVI   |  |  |  |
| Post-TAVI   |  |  |  |
| Healthy   |  |  |  |
| **TR 163-21** | Mode 1: $\Phi_{1}$ | Mode 2: $\Phi_{2}$ | Mode 3: $\Phi_{3}$ |
| Pre-TAVI   |  |  |  |
| Post-TAVI   |  |  |  |
| Healthy   |  |  |  |
| **TR 178-22** | Mode 1: $\Phi_{1}$ | Mode 2: $\Phi_{2}$ | Mode 3: $\Phi_{3}$ |
| Pre-TAVI   |  |  |  |
| Post-TAVI   |  |  |  |
| Healthy   |  |  |  |

**S-V: Temporal normalized POD coefficients *b(t)***

Supplementary Figure 1 shows the temporal evolution of the normalized POD coefficients for modes 1-4 across all patients. Mode 1 consistently captures the dominant temporal dynamics during systole, while the other modes represent smaller contributions. The comparison across states allows assessment of how treatment influences the temporal distribution of energy among modes relative to the healthy reference.

**Supplementary Figure 1** Temporal normalized POD coefficients **b**(t) for modes 1-4, plotted per patient in pre-intervention (left column), post-intervention (middle column) and healthy (right column) states. Mode 1 (red) explains the largest proportion of variance, while the remaining modes 2-4 (blue, green, purple, respectively) account for progressively smaller average contributions.

**S-VI: POD Reconstruction Error Metrics**

Supplementary Figure 2 summarizes the accuracy of the POD-based flow reconstructions using the first four POD modes. Reconstruction quality was assessed using four complementary error metrics: the Pearson coefficient (R^2^), root mean squared error (RMSE), relative error and absolute deviation. Across all patients and states, R^2^ values remained consistently high, indicating strong agreement between reconstructed and original velocity fields. RMSE values were low with small relative errors and absolute deviation magnitudes, demonstrating reconstruction performance. These results confirm that the chosen number of POD modes provides a reliable representation of the flow field across conditions, with no major differences between different states.

**Supplementary Figure 2** Error analysis of POD-based flow reconstruction. Each point represents a single timestep for one patient in a given state. Error metrics include the Pearson coefficient (R^2^), root mean squared error (RMSE), relative difference and absolute deviation across all spatial velocity points. Boxplots indicate distribution across patients, with median values reported below each group.

References

[1]	Faghih, Mohammad M. and Sharp, M. Keith. “Modeling and prediction of flow-induced hemolysis: a review.” *Biomechanics and modeling in mechanobiology* Vol. 18 No. 4 (2019): pp. 845–881. DOI 10.1007/s10237-019-01137-1.

[2]	Konnigk, Lucas, Torner, Benjamin, Bruschewski, Martin, Grundmann, Sven, and Wurm, Frank-Hendrik. “Equivalent Scalar Stress Formulation Taking into Account Non-Resolved Turbulent Scales.” *Cardiovascular engineering and technology* Vol. 12 No. 3 (2021): pp. 251–272. DOI 10.1007/s13239-021-00526-x.

[3]	Morbiducci, Umberto, Ponzini, Raffaele, Rizzo, Giovanna, Cadioli, Marcello, Esposito, Antonio, Cobelli, Francesco de, Del Maschio, Alessandro, Montevecchi, Franco Maria, and Redaelli, Alberto. “In vivo quantification of helical blood flow in human aorta by time-resolved three-dimensional cine phase contrast magnetic resonance imaging.” *Annals of biomedical engineering* Vol. 37 No. 3 (2009): pp. 516–531. DOI 10.1007/s10439-008-9609-6.

[4]	Zhou, Hai-Hong, Tang, Yu-Long, Xu, Tian-Hao, and Cheng, Bin. “C-reactive protein: structure, function, regulation, and role in clinical diseases.” *Frontiers in immunology* Vol. 15 (2024): p. 1425168. DOI 10.3389/fimmu.2024.1425168.

[5]	Link, Jeffrey J., Rohatgi, Anand, and Lemos, James A. de. “HDL cholesterol: physiology, pathophysiology, and management.” *Current problems in cardiology* Vol. 32 No. 5 (2007): pp. 268–314. DOI 10.1016/j.cpcardiol.2007.01.004.

[6]	Yi, Sang-Wook, Yi, Jee-Jeon, and Ohrr, Heechoul. “Total cholesterol and all-cause mortality by sex and age: a prospective cohort study among 12.8 million adults.” *Scientific reports* Vol. 9 No. 1 (2019): p. 1596. DOI 10.1038/s41598-018-38461-y.

[7]	Nordestgaard, Børge G., Chapman, M. John, Ray, Kausik, Borén, Jan, Andreotti, Felicita, Watts, Gerald F., Ginsberg, Henry, Amarenco, Pierre, Catapano, Alberico, Descamps, Olivier S., Fisher, Edward, Kovanen, Petri T., Kuivenhoven, Jan Albert, Lesnik, Philippe, Masana, Luis, Reiner, Zeljko, Taskinen, Marja-Riitta, Tokgözoglu, Lale, and Tybjærg-Hansen, Anne. “Lipoprotein(a) as a cardiovascular risk factor: current status.” *European heart journal* Vol. 31 No. 23 (2010): pp. 2844–2853. DOI 10.1093/eurheartj/ehq386.

[8]	Melzer, Susanne, Zachariae, Silke, Bocsi, Jozsef, Engel, Christoph, Löffler, Markus, and Tárnok, Attila. “Reference intervals for leukocyte subsets in adults: Results from a population-based study using 10-color flow cytometry.” *Cytometry. Part B, Clinical cytometry* Vol. 88 No. 4 (2015): pp. 270–281. DOI 10.1002/cyto.b.21234.

[9]	Said, Elias A., Al-Reesi, Iman, Al-Shizawi, Nawal, Jaju, Sanjay, Al-Balushi, Mohammed S., Koh, Crystal Y., Al-Jabri, Ali A., and Jeyaseelan, Lakshmanan. “Defining IL-6 levels in healthy individuals: A meta-analysis.” *Journal of medical virology* Vol. 93 No. 6 (2021): pp. 3915–3924. DOI 10.1002/jmv.26654.

[10]	Welsh, Paul, Campbell, Ross T., Mooney, Leanne, Kimenai, Dorien M., Hayward, Caroline, Campbell, Archie, Porteous, David, Mills, Nicholas L., Lang, Ninian N., Petrie, Mark C., Januzzi, James L., McMurray, John J. V., and Sattar, Naveed. “Reference Ranges for NT-proBNP (N-Terminal Pro-B-Type Natriuretic Peptide) and Risk Factors for Higher NT-proBNP Concentrations in a Large General Population Cohort.” *Circulation. Heart failure* Vol. 15 No. 10 (2022): e009427. DOI 10.1161/CIRCHEARTFAILURE.121.009427.
